# Healthcare costs of cancer among children, adolescents, and young adults: A scoping review

**DOI:** 10.1002/cam4.6925

**Published:** 2024-01-12

**Authors:** Doreen Nabukalu, Louisa G. Gordon, John Lowe, Katharina M. D. Merollini

**Affiliations:** ^1^ School of Health University of the Sunshine Coast Sippy Downs Queensland Australia; ^2^ Population Health Program QIMR Berghofer Medical Research Institute Herston Queensland Australia; ^3^ School of Nursing Queensland University of Technology Kelvin Grove Queensland Australia; ^4^ School of Public Health The University of Queensland Herston Queensland Australia; ^5^ Sunshine Coast Health Institute Sunshine Coast University Hospital Birtinya Queensland Australia

**Keywords:** adolescent, cancer, child, healthcare costs, neoplasms, oncology, young adult

## Abstract

**Objective:**

To collate and critically review international evidence on the direct health system costs of children and adolescents and young adults (AYA) with cancer.

**Methods:**

We conducted searches in PubMed, MEDLINE, CINAHL, and Scopus. Articles were limited to studies involving people aged 0–39 years at cancer diagnosis and published from 2012 to 2022. Two reviewers screened the articles and evaluated the studies using the Consolidated Health Economic Evaluation Reporting Standards checklist. The reviewers synthesized the findings using a narrative approach and presented the costs in 2022 US dollars for comparability.

**Results:**

Overall, the mean healthcare costs for all cancers in the 5 years post diagnosis ranged from US$36,670 among children in Korea to US$127,946 among AYA in the USA.

During the first year, the mean costs among children 0–14 years ranged from US$34,953 in Chile to over US$130,000 in Canada. These were higher than the costs for AYA, estimated at US$61,855 in Canada. At the end of life, the mean costs were estimated at over US$300,000 among children and US$235,265 among adolescents in Canada.

Leukemia was the most expensive cancer type, estimated at US$50,133 in Chile, to US$152,533 among children in Canada. Overall, more than a third of the total cost is related to hospitalizations. All the included studies were of good quality.

**Conclusions:**

Healthcare costs associated with cancer are substantial among children, and AYA. More research is needed on the cost of cancer in low‐ and middle‐income countries and harmonization of costs across countries.

## 1 BACKGROUND

Cancer incidence is rising among children^1^, ^2^ and adolescents and young adults (AYA),^3^ which presents a significant disease and economic burden across the world.^4^ Globally, in 2020, there were an estimated 204,665 new cases of childhood cancers in children aged 0–14 years and 1,233,225 new cases in AYA aged 15–39 years.^5^ These were mainly leukemias, and brain and central nervous system (CNS) tumors among children, and breast and thyroid cancers among AYA.^5^ In high‐income countries, most of these cancers are treated successfully and yield a high 5‐year survival rate of over 80%^6^, ^7^ for children and AYA with cancer.

A significant amount of healthcare resources, including medication, imaging, pathology, radiology, and specialists,^8^, ^9^, ^10^, ^11^ are needed for diagnosis and treatment of cancer, and to manage its side effects. In addition, the need for surveillance of secondary cancers or cancer relapse, and psychological care creates a significant demand for services after active treatment.^12^

The costs of providing healthcare for children and AYA with cancer are not well understood. Several studies and reviews on the costs of cancers in adult populations have been conducted^13^, ^14^, ^15^, ^16^, ^17^, ^18^, ^19^, ^20^ and highlight the high direct costs in adult cancer populations compared to those without cancer. However, parallel evidence is lacking for those diagnosed with cancer as children and AYA.

In this study, we performed a scoping review to evaluate the current literature on the healthcare costs of all cancers in children and AYA from a healthcare provider perspective. The evidence generated is important in understanding the health system cost implications of cancer, which is a key factor in informing government health policy, resource allocation and health services management. In addition, this evidence can contribute to economic evaluations concerned with allocating healthcare resources for childhood and AYA cancers.

## 2 METHODS

This review followed the Joanna Briggs Institute (JBI) methodological guidance for conducting scoping reviews^21^ and used dedicated JBI software. We developed a protocol outlining a detailed search strategy and inclusion criteria. The findings of this review followed the Preferred Reporting Items for Systematic Reviews and Meta‐Analyses extension for Scoping Reviews (PRISMA‐ScR).^22^

### 2.1 Eligibility criteria

English‐language studies published from January 2012 to September 2022 were included and confined to this time frame to capture healthcare costs that reflected current clinical practices. The studies had to be quantitative and focused on cancer in children, adolescents, or young adults (defined as being aged 0–39 years). Healthcare costs were a primary or secondary outcome. We defined direct healthcare costs as expenses related to cancer treatment and care paid by the government and providers. Qualitative studies, protocols, reviews, commentaries, and conference abstracts were not included. We did not search for articles in the gray literature.

### 2.2 Information sources and literature search strategy

The search for relevant articles was conducted in four databases: MEDLINE, PubMed, CINAHL, and Scopus. First, an initial limited search of PubMed was undertaken. We created a search strategy (Data S1) with the help of a medical librarian using Medical Subject Headings (MeSH) and article keywords. The strategy was then adapted to three other databases, including MEDLINE (Web of Science), CINAHL (EBSCO), and Scopus.

### 2.3 Selection of sources of evidence

We collected and saved all the citations from various databases in EndNote X9 (Clarivate Analytics, PA, USA). We removed duplicate citations and imported the remainder into the JBI System for the Unified Management, Assessment, and Review.^21^ Two reviewers (DN and KM) independently screened the titles and abstracts of the retrieved articles. We checked all references of selected articles for other relevant ones, which we retrieved and added for full‐text review. The reasons for exclusion were recorded in the JBI system. The two reviewers discussed any disagreements that arose over the eligibility of the article and came to a consensus for inclusion or exclusion.

### 2.4 Data charting process and data items

The Excel™ database recorded study information, such as authors, year of publication, country, sample size, perspective, cost estimates, cost components (such as hospitalization, emergency department presentations, pharmaceutical use.), and study funding sources.

Data on the cost outcomes were charted in the currency reported in the respective studies and using the web‐based CCEMG—EPPI‐Centre Cost Converter,^23^ adjusted to 2022 prices and converted to a common currency, United States Dollars (USD).

### 2.5 Critical appraisal and quality assessment of sources of evidence

We appraised the quality of study reporting using the standardized Consolidated Health Economic Evaluation Reporting Standards (CHEERs) checklist.^24^ Given most of our studies were cost‐of‐illness studies, and CHEERs covers full economic evaluations, we limited our appraisal to 13 out of 23 items of the CHEERs checklist that were deemed relevant.

### 2.6 Synthesis of results

Studies in this review had a diverse range of cost components or categories and methods across the different age groups which precluded performing a meta‐analysis. Therefore, we performed a narrative synthesis and summarized the study characteristics, costing methods, study perspective, healthcare cost outcomes, and cost components.

## 3 RESULTS

### 3.1 Selection of studies

**FIGURE 1:**
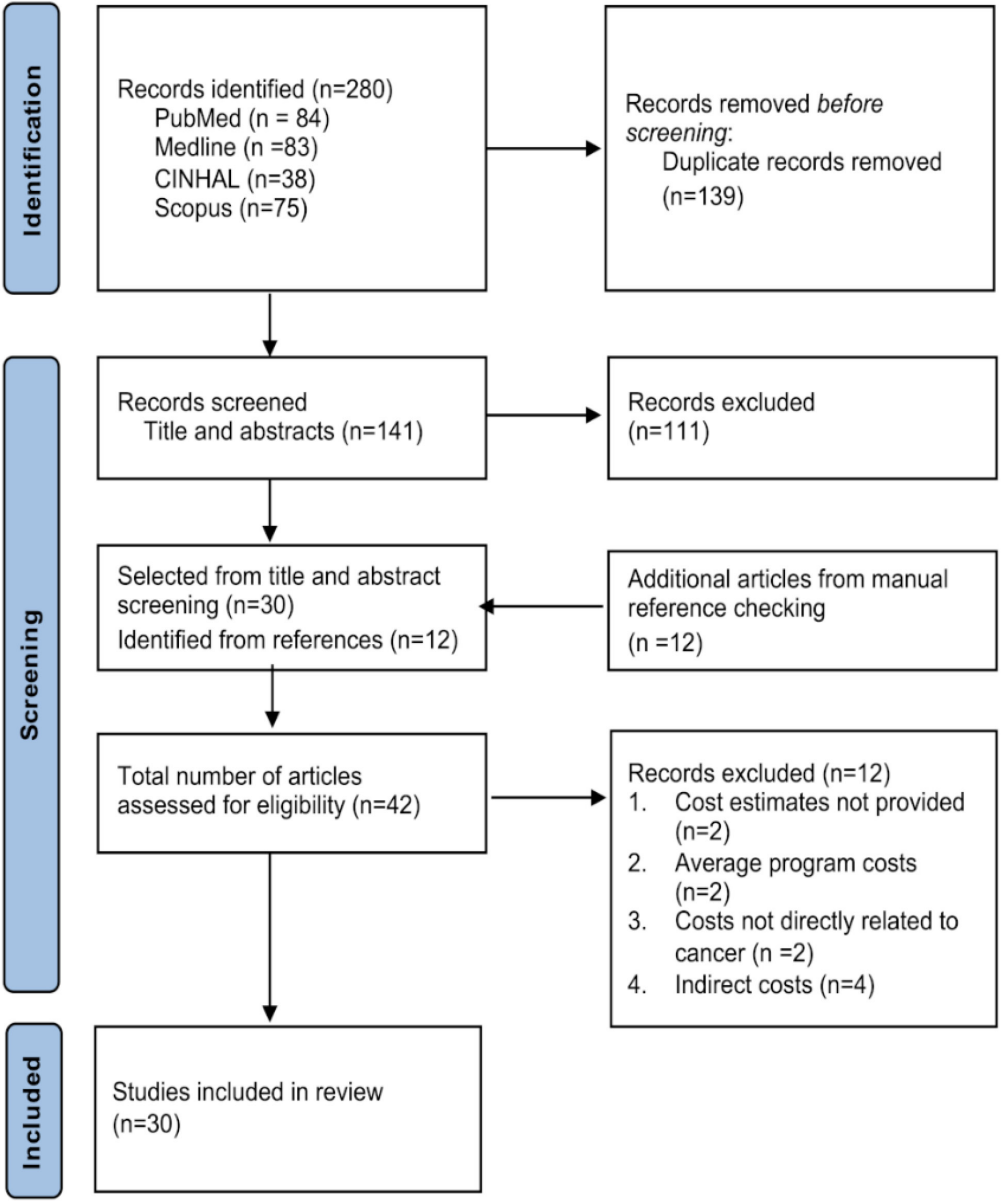
Flow diagram of search and study selection process.

Our search yielded 141 articles after the removal of duplicates. After screening the titles, abstracts, and full texts against the inclusion criteria, 30 articles were eligible for inclusion (Figure 1).

### 3.2 Study settings

The selected studies were conducted across 13 countries, predominantly in the United States (*n* = 12, 40%)^25^, ^26^, ^27^, ^28^, ^29^, ^30^, ^31^, ^32^, ^33^, ^34^, ^35^, ^36^ and Canada (*n* = 5, 17%).^37^, ^38^, ^39^, ^40^, ^41^ Australia^42^, ^43^ and the Netherlands^44^, ^45^ reported two studies each while nine other countries including France, Chile, Korea, India, Mexico, Spain, Egypt, Thailand, and China had one study (Table 1, Table S2). A total of five (*n* = 5,17%) studies were in middle‐income countries with the majority (*n* = 25, 83%) in high‐income countries. None of the studies were from a low‐income country (Table S2).

**TABLE 1:** Characteristics of the *N* = 30 included studies.

First author, year	Country	Study design	Study population	Cancer site	Cost outcome	Perspective	Cost calculation	Data source	Findings	Currency/reference year
Audino 2013	USA	Retrospective cohort	Pediatric & AYA patients (0–39 years)	Bone sarcomas	Health care costs	Healthcare system perspective	Bottom‐up	Pediatric Health Information System for bone sarcoma admissions	Higher pharmaceutical costs for AYA compared to children ($18,124 vs. $13,637)	USD
Abdelhadi 2022	USA	Panel survey	AYA diagnosed with cancer (15–39 years)	All cancers except non‐melanoma skin cancers	Medical expenditures	Healthcare system perspective	Bottom‐up	Medical Expenditure Panel Survey (MEPS)	Average annual medical expenditures of $5324 for AYA cancer survivors without psychological distress in. Additional $4415 with psychological distress	USD/2016
Abdelhadi 2022	USA	Panel survey	AYA cancer survivors (15–39 years)	All cancers except non‐melanoma skin cancers	Medical expenditures	Healthcare system perspective	Bottom‐up	Medical Expenditure Panel Survey (MEPS)	Average annual medical expenditures of $5468 for AYA cancer survivors without chronic conditions. Additional $2777 for those with chronic conditions	USD/2016
Bejarano‐Quisoboni 2022	France	Retrospective cohort	Five‐year childhood cancer survivors (0–21 years)	All cancers except leukemia	Direct healthcare expenditure	Payer perspective	Bottom‐up	French Childhood Cancer Survivors Study Cohort, cancer registry	The mean annual amount of healthcare expenditures was €4255	Euros/2015
Benedict 2021	USA	Retrospective cohort	Childhood cancer Survivors	All cancers	Survivorship care cost	Healthcare system perspective	Bottom‐up	Financial records of the Northwell Health System	The average cost was $1211.44 for the adherent group and $2469.84 for the non‐adherent group	USD/2012
Borrescio‐Higa 2018	Chile	Retrospective cohort	Children with cancer (0–18)	All cancers	Total cost and out‐of‐pocket spending by patients	Societal perspective	Bottom‐up	Administrative Records of Children with private insurance	Children with cancer had high annual medical costs, USD 32,287 on average	USD/2018
Chae 2020	Korea	Retrospective cohort	Cancer patients (0–17 years)	All cancers	Total medical cost	National Health Insurance Service	Bottom‐up	Korean National Health Insurance Claims Database	The average total medical cost per patient is 32,157 United States Dollars	USD/2015
Cheng Brian & Wangmo 2020	USA	Retrospective cohort	Children with cancer (<18 years)	Solid organ or blood cancer	Palliative care costs	Healthcare system perspective	Bottom‐up	The National Inpatient Sample (NIS)	The mean total costs of care for admissions with versus without PC utilization were $72,364 (95% CI: $50,660,94,068) versus $132,036 ($120,117, 143,955)	USD/2014
de Oliveira 2017	Canada	Retrospective cohort	Patients with cancer (91 days to 19 years)	All cancers	Health resource‐specific costs	Public payer perspective	Bottom‐up	Using linked administrative health care records	Mean net postdiagnosis costs were $136,413 and $62,326 for children & adolescents, respectively	CAD/2012
Oliveira 2017	Canada	Retrospective cohort	Children and AYA newly diagnosed (0–19.9 years)	All cancers	Healthcare costs by phase of cancer care	Public payer perspective	Bottom‐up	Using linked administrative health care records	Costs for initial, continuing, and final phases were $138,161, $15,756, and $316,303 per 360 days for children, and $62,919, $7071, and $242,008 for adolescents	CAD/2012
Ghatak 2016	India	Cross sectional analysis	Children with ALL (1–12 years)	Acute lymphoblastic leukemia	Medical expenditures	Societal perspective	Bottom‐up	Patient cost sheet maintained for 1 month	The medical expenditure amounted to $524 IQR ($395–$777)	USD/2013
Gupta 2021	Canada	Retrospective cohort	All children with Acute lymphoblastic leukemia (<18 years)	Acute lymphoblastic leukemia	Healthcare utilization costs	Healthcare system perspective	Bottom‐up	Pediatric Oncology Group of Ontario Networked Information System	The mean total health care cost in the first 5 years after initial diagnosis was $238,800 among COG patients compared with $333,870 among DFCI patients	CAD/2018
Guy 2014	USA	Panel survey	Adolescent and young adult cancer survivors (15–39 at cancer diagnosis)	All cancers	Direct medical costs	Societal perspective	Bottom‐up	Medical Expenditure Panel Survey data	Excess annual medical expenditures for AYA cancer survivors were $3170 per person	USD/2011
Haeusler 2018	Australia	External validation study	Children with FN (0–19 years)	All cancers	Hospitalization costs	Healthcare system perspective	Bottom‐up	Electronic databases of the Royal Children's Hospital (RCH), Melbourne	The total average cost for low‐risk episodes was significantly less than high‐risk episodes by AUD 10,758	AUD/2018
Jaime‐Pérez 2017	Mexico	Retrospective cohort	Pediatric patients (0–15 years)	Acute Lymphoblastic Leukemia	Hospitalization rate & cost	Healthcare system perspective	Top‐down	Electronic and paper‐based medical records	3038 USD for standard‐risk patients. (Per patient per year (PPPY))	USD/2016
Kloos 2019	Netherlands	Cost effective analysis	Children with acute lymphoblastic leukemia (0–12 years)	Acute Lymphoblastic Leukemia	Total treatment costs for two protocols	Dutch hospital perspective	Bottom‐up	Hospital medical files	The mean total costs per patient were $40,925 without a hypersensitivity reaction to PEG asparaginase, $175,632 when switched to Erwinia asparaginase, and $21,190 if asparaginase therapy permanently stopped	USD
Lekshminarayanan 2018	USA	Retrospective cohort	Pediatric inpatient encounters of FN (0–19 years)	All cancers	Cost for inpatient resource use	Healthcare system perspective	Bottom‐up	National (Nationwide) Inpatient Sample (NIS) database	The median cost of hospitalization increased from $8771 (2007) to $11,202 (2014)	USD/2014
McBride 2020	Canada	Retrospective cohort	Cancer patients diagnosed at (<15 years)	All cancers	Direct medical costs by phase of care in British Columbia and Ontario	Healthcare system perspective	Bottom‐up	Population‐based healthcare administrative data	For all cancers combined, mean net costs in ON were higher than those in BC	CAD/2012
McGrady 2017	USA	Retrospective cohort	AYAs with cancer receiving psychology services (15–35 years)	All cancers	Psychology service use and spending on hospital care	Healthcare system perspective	Bottom‐up	Electronic medical records	Spending was higher among AYAs with leukemia ($1,149,589.04) than AYAs with lymphoma ($490,367.46)	USD/2015
Mueller 2017	USA	Cross sectional analysis	Children enrolled on medicaid (0–18 years)	All cancers	Healthcare utilization and spending	Healthcare system perspective	Bottom‐up	Healthcare Cost and Utilization Project's (HCUP), Kids' Inpatient Database (KID)	Spending for children with cancer was $3706 overall and $2323 for hospital care	USD/2015
Mueller 2016	USA	Cross sectional analysis	Pediatric patients who had a discharge for FN (0–19 years)	All cancers	Hospital charges	Healthcare system perspective	Bottom‐up	2014 Truven Marketscan Medicaid Database	The mean hospital charge was $65,536 for FN discharges among pediatric patients with cancer	USD/2015
Nathan 2019	Canada	Retrospective cohort	All ontario adolescents (15.0–17.9 years)	All cancers except non‐melanoma skin cancers	Healthcare utilization and costs	The payer perspective	Bottom‐up	Ontario Cancer Registry & the Pediatric Oncology Group of Ontario's Networked Information System (POGONIS)	For all diagnoses, median initial phase costs were higher in pediatric than adult institutions (e.g., leukemia: $153,926 vs. $102,418 per year)	CAD/2012
Maria 2020	Spain	Cost‐effective analysis	Pediatric and AYA (1–25 years)	Acute lymphoblastic leukemia	Costs of pharmacological treatments and health resource use	Health System perspective	Mixed methods	Other study databases ELIANA, ENSIGN & B2101J study	Higher cost (€ 258,378) for tisagenlecleucel compared to salvage chemotherapy	Euros/2018
Kaul 2016	USA	Retrospective cohort	Newly diagnosed cancer patients (1–26 years)	Acute lymphoblastic leukemia	Hospitalization costs	Hospital perspective	Top‐down	System‐wide cancer registry and enterprise data warehouse	The average or per‐patient first‐year hospitalization costs rose from $24,197 in 1998 to $37,924 in 2012	USD/2012
Soliman 2021	Egypt	Retrospective cohort	Children with cancer (0–18 years)	All cancers	Hospital resource use and costs	Healthcare provider perspective	Bottom‐up	Costing data at the costing/billing database at the Oracle system	For all cancers combined, median costs were $14,774 at 1 year and $19,799 at 3 years post diagnosis	USD/2019
Sruamsiri 2020	Thailand	Cost‐analysis	Children with incident ALL (0–14 years)	Acute lymphoblastic leukemia	Chemotherapy costs for ALL	Health system perspective	Bottom‐up (Using price and volume data)	Pharmaceutical Information Center, Ministry of Public Health, Thailand	Essential chemotherapy to treat all children diagnosed with ALL in Thailand in 2017 would cost US$ 814,952 (US$ 1,365,422 for diagnosed and undiagnosed children)	USD/2017
Tan 2022	Australia	Retrospective cohort	Childhood cancer survivors. (0–19 years)	All cancers	Hospital and emergency department costs	Payer perspective	Bottom‐up approach	NSW Central Cancer Registry, and National Hospital Cost Data Collection estimates	The estimated median annual cost of hospitalization in the first year after diagnosis was A$88,964 for patients diagnosed at age 0 to 14 years and A$23,384 for those diagnosed at age 15 to 17 years	AUD/2018
Taparra 2022	USA	Retrospective cohort	AYA cancer patients (15–39 years)	All cancers	Direct costs	Health System perspective	Bottom‐up	Cancer center registry at a community‐based hospital system	Median total costs per patient were $123 K	USD/2020
Tong 2013	The Netherlands	Cost‐analysis	Children with acute lymphoblastic leukemia (<18 years)	Acute lymphoblastic leukemia	Chemotherapy costs	Health System perspective	Bottom‐up	Data on volumes were adapted from hospital electronic databases and medical files	The total costs of the intensification course of 30 weeks were $57,893 in patients without PEGasparaginase allergy (*n* = 64). The costs were significantly higher ($113,558) in case of allergy (*n* = 20)	USD
Zhou 2021	China	Retrospective cohort	Inpatients with malignant tumors (0–18 years)	All cancers	Treatment costs	Health System perspective	Bottom‐up	Inpatient electronic health record (EHR) data	The acute myeloid leukemia group had the highest total cost, slightly lower than that of CNS tumors in the solid tumor group by 44,731.37 (36,375.47–64,140.93) RMB	USD

Abbreviations: HCUP, Healthcare Cost and Utilization Project's; KID, Kids' Inpatient Database; MEPS, Medical Expenditure Panel Survey; NIS, National Inpatient Sample.

### 3.3 Study designs and data sources

Most studies were observational by design. These included retrospective cohorts (*n* = 19, 63%), panel surveys (*n* = 3, 10%), cross‐sectional analysis (*n* = 3, 10%), two cost‐effectiveness analyses,^44^, ^46^ one external validation study^42^ and two cost analyses^45^, ^47^ (Table 1, Table S1).

### 3.4 Participant age group

More than half of the studies (*n* = 16, 53%) reported costs for both children and adolescents 0–19 years. Other studies were exclusive to children 0 to 14 years (*n* = 4, 13%),^39^, ^47^, ^48^, ^49^ AYA aged 15 to 39 years (*n* = 5, 17%),^25^, ^26^, ^30^, ^33^, ^36^ or adolescents 15 to 19 years (*n* = 1, 3%).^40^ Four studies (*n* = 4, 13%) covered the whole spectrum of children and AYA aged 0–39 years^27^, ^28^, ^31^, ^46^ (Table 1, Table S1).

### 3.5 Participant cancer types

Regarding the type of cancer, more than half of the studies (*n* = 16, 53%) quantified costs for all cancer types and three studies excluded melanoma skin cancers (*n* = 3, 10%).^25^, ^26^, ^40^ Other studies covered one type of cancer, such as acute lymphoblastic leukemia (*n* = 8, 27%) and bone sarcomas (*n* = 1, 3%)^27^ (Table 1, Table S1).

### 3.6 Sample size

The study sample sizes referred to either the number of patients or episodes of care. Patient sample sizes ranged from 50^49^ to 88,329^30^ with a mid‐range of 1376. The number of episodes of care, such as hospital admissions, ranged from 3853^50^ to 6,675,222^35^ in four studies.^29^, ^32^, ^34^, ^50^ Two studies reported both the numbers of patients and episodes of care in the sample size description^42^, ^48^ (Table 2, Table S1).

**TABLE 2:** Health Care Costs per Phase of Diagnosis in 2022 US Dollars.

Study	Country	Sample size	Units of measure	COSTS per phase of diagnosis in 2022 US Dollars				
				Pre‐diagnosis	Initial/1 year post_ diagnosis	Continuing care	End‐of‐life care	Overall costs
TOTAL MEDICAL COSTS								
Children (0–14 years) and Adolescents (0–19 years)								
*All cancers*								
Mueller 2017	USA	5905 children with cancer	Per member per month			$4226		
de Oliveira 2017	Canada	4396 patients (91 days to 14 years) & 2329 patients (15 to 19 years)	Mean total costs	$6116	$133,325			
Oliveira 2017	Canada	4606 children & 2443 adolescents	Mean total costs per weighted case	$6426	$135,029	$16,253	$309,203	
McBride 2020	Canada (BC)	1503 cases in BC	Mean net total cost per patient	$4838	$96,487	$13,175	$300,938	
McBride 2020	Canada (Ontario)	1503 cases in Ontario	Mean net total cost per patient	$6426	$135,029	$16,253	$306,269	
Chae 2020	Korea	7317 patients	Mean total cost					$36,670
Borrescio‐Higa 2018	Chile	3853 observations	Average annual medical costs in 2018		$34,953			
Zhou 2021	China		Median total cost					$5315–$8696
*Acute lymphoblastic leukemia*								
W.H. Tong 2013	Netherlands	84 subjects	Mean total treatment costs					$66,019 to $129,496
Gupta 2021	Canada	802 children	Mean total Chemotherapy costs					$214,113 to $299,354
Sruamsiri 2020	Thailand	318 children	Total chemotherapy/chemoprotective agent costs					$902,141 to $1,511,504
Adolescents (15–19) years								
*All cancers*								
de Oliveira 2017	Canada	4396 patients (91 days to 14 years) & 2329 patients (15 to 19 years)	Mean total costs	$5585	$61,251			
Oliveira 2017	Canada	4606 children and 2443 adolescents	Mean total costs per weighted case	$7128	$61,855	$7749	$235,265	
AYA (15–39) years								
*All cancers*								
Taparra 2022	USA	388 AYA patient	Median total costs per patient	$1459	$124,091			$127,946
Children, adolescents and young adults (0–39 years)								
*Acute lymphoblastic leukemia*								
Maria 2020	Spain	Not clear	Total chemotherapy costs					$99,047 to $357,425
HOSPITALIZATION COSTS								
Children (0–14 years) and Adolescents (0–19 years)								
*All cancers*								
Tan 2022	Australia	2966 patients	Mean total	$294	$86,799	$13,072		$122,567
Soliman 2021	Egypt	8886 children	Median total costs per patient		$15,715	$21,059		
*Acute lymphoblastic leukemia*								
Jaime‐Pérez 2017	Mexico	101 patients & 449 hospital admissions	Total hospitalization costs per patient per year					$5486
*Palliative care*								
Cheng and Wangmo 2019	USA	10,960 hospitalizations	Mean total cost for admissions					$83,404
Adolescents (15–19) years								
*All cancers*								
Tan 2022	Australia	2966 patients	Mean total	$175	$47,487	$9427		$67,982
Children, adolescents and young adults (0–39 years)								
*Acute lymphoblastic leukemia*								
Kaul 2016	USA	505 patients	Average or per‐patient hospitalization		$45,318			
SURVIVORSHIP CARE								
Children (0–14 years) and Adolescents (0–19 years)								
Benedict 2021	USA	286 patients (3‐years post diagnosis)	Average cost of recommended follow‐up care					$1448 to $2951
Bejarano‐Quisoboni 2022	France	5319 (5‐years post diagnosis)	Mean annual expenditure					$5914
AYA (15–39) years								
Guy 2014	USA	1464 AYA (All with history of cancer)	Annual expenditure per person					$9033
Abdelhadi 2022	USA	1757 AYA (All with history of cancer)	Mean annual expenditure					$6006
Abdelhadi 2022	USA	2326 AYA (All with history of cancer)	Mean annual expenditure					$6168

### 3.7 Data sources

Electronic administrative data and billing records were the predominant sources of resource and cost data for 26 studies (*n* = 26, 87%) (Table 1, Table S1). Three studies (*n* = 3, 10%) used data from the USA based Medical Expenditure Panel Survey.^25^, ^26^, ^30^ One study used data from the Healthcare Cost and Utilization Project^34^ and one study used prospective cross‐sectional survey data^49^ (Table 1, Table S1).

### 3.8 Quality of reporting of the costing methods

The quality of the studies was good overall, with sufficient information on the population and context of the study, clear descriptions of how costs were valued, and suitable measures used to summarize the study outcomes. Most studies (*n* = 26, 86%) reported over 80% of the items on the CHEERS checklist (Table S6). The costing perspective was the least reported item with 12 studies (*n* = 12, 40%) specifying their costing perspective.^28^, ^37^, ^38^, ^40^, ^41^, ^43^, ^44^, ^46^, ^49^, ^51^, ^52^, ^53^ However, based on the composition of the cost units, half of the studies (*n* = 12, 50%) employed a health system perspective, 12 studies used a payer's perspective and three studies had a societal perspective (Table 1). 50% of the studies (*n* = 15) were funded by national health funding bodies, one^46^ by a pharmaceutical company *Novartis Farmacéutica*, three studies had no funding and the remainder (*n* = 12, 40%) did not mention their funding sources.

### 3.9 Direct health system costs

Four studies assessed the cost of cancer throughout its entire journey from diagnosis to end of life,^39^, ^40^, ^41^, ^48^ while two studies focused on the 5‐year diagnosis period.^36^, ^43^ Five studies were exclusive to long‐term cancer survivors, two of which were 3‐ and 5‐year post cancer diagnosis.^25^, ^26^, ^28^, ^30^, ^54^ In the rest of the studies, participants either were still with cancer,^27^, ^29^, ^32^, ^34^, ^35^, ^42^, ^44^, ^45^, ^46^, ^47^, ^55^ within 1‐year post diagnosis^37^, ^49^, ^50^ or up to 3‐year post diagnosis.^31^, ^33^, ^53^

Cost estimates were limited in time or specific to a particular care phase, cancer type, type of cost (e.g., hospital use and medication costs). High costs were seen in pediatric patients, those with leukemia, and during the first year of cancer diagnosis and end of life.

Based on selected studies, total healthcare costs for cancer per person varied from US$36,670 in Korea^51^ to US$127,946 in the USA.^36^

### 3.10 Costs related to the first year of cancer diagnosis

The mean healthcare cost for children in the first year of cancer diagnosis ranged between US$34,953 in Chile^50^ to over US$130,000 in Canada^37^, ^39^, ^41^ (Table 2). Among adolescents in Canada, the highest mean healthcare cost was US$61,855 in the first year of diagnosis.^41^ In Australia, hospitalization costs followed a similar pattern, with children costing a median annual cost US$86,799 and adolescents costing US$47,487 per patient in their first year of cancer diagnosis^43^ (Table 2). For AYA, a study in the USA estimated the median annual expenditure post‐diagnosis was US$12,4091 post‐diagnosis^36^ (Table 2).

### 3.11 Costs related to ongoing care and survivorship care

Like the first year of cancer diagnosis, total healthcare costs and hospitalizations costs during continuing care were higher for children compared to adolescents in both Canada and Australia. In Canada, mean annual costs for children and adolescents were US$16,253 and US$7749, respectively, while hospitalization costs during continuing care were US$13,072 for children and US$9427 for adolescents in Australia.

Three studies estimated the yearly costs of cancer survivorship beyond 5 years. On average, these ranged from US$1448 in the USA^28^ to US$5914 among long‐term childhood cancer survivors in France annually^54^ (Table 2). Costs for cancer survivors were higher for AYA in the USA, estimated at US$9033 per year.^54^

### 3.12 Costs related to end‐of‐life care and palliative care

Compared to other phases of care, costs were generally higher during the end‐of‐life care phase, commonly referred to as the last 12 months of life. The cost of end‐of‐life care is higher for children than for adolescents in Canada, at over US$300,000^39^, ^41^ and US$235,265,^41^ respectively. In a US study of children with cancer and a high in hospital mortality, those receiving palliative care had lower hospital costs compared to those not receiving palliative care estimated at US$83,403 and S$152,179, respectively^29^ (Table 2).

### 3.13 Cancer‐specific costs

In all studies, leukemia had the highest cost compared to other cancers for children and adolescents. For children, costs specific to leukemia were estimated at US$50,133 in Chile^50^ to US$152,533 in Canada.^37^, ^41^ Studies in Canada estimated the cost of leukemia ranging from US$149,045 to US$166,670 among adolescents.^40^, ^41^ Other costly cancers included brain and central nervous system (CNS) tumors, bone and articular cartilage, and non‐Hodgkin lymphoma (Table S3).

Four studies exclusively estimated the cost of different treatment protocols and therapies for acute lymphoblastic leukemia (ALL).^38^, ^45^, ^46^, ^47^ These included treatment with asparaginase preparations in the Netherlands with mean costs ranging from US$66,019 to US$129,497^45^ and other treatment protocols in Canada for chemotherapy ranging from US$214,113 to US$299,354^38^ (Table 2).

### 3.14 Health service components of the costs

**FIGURE 2:**
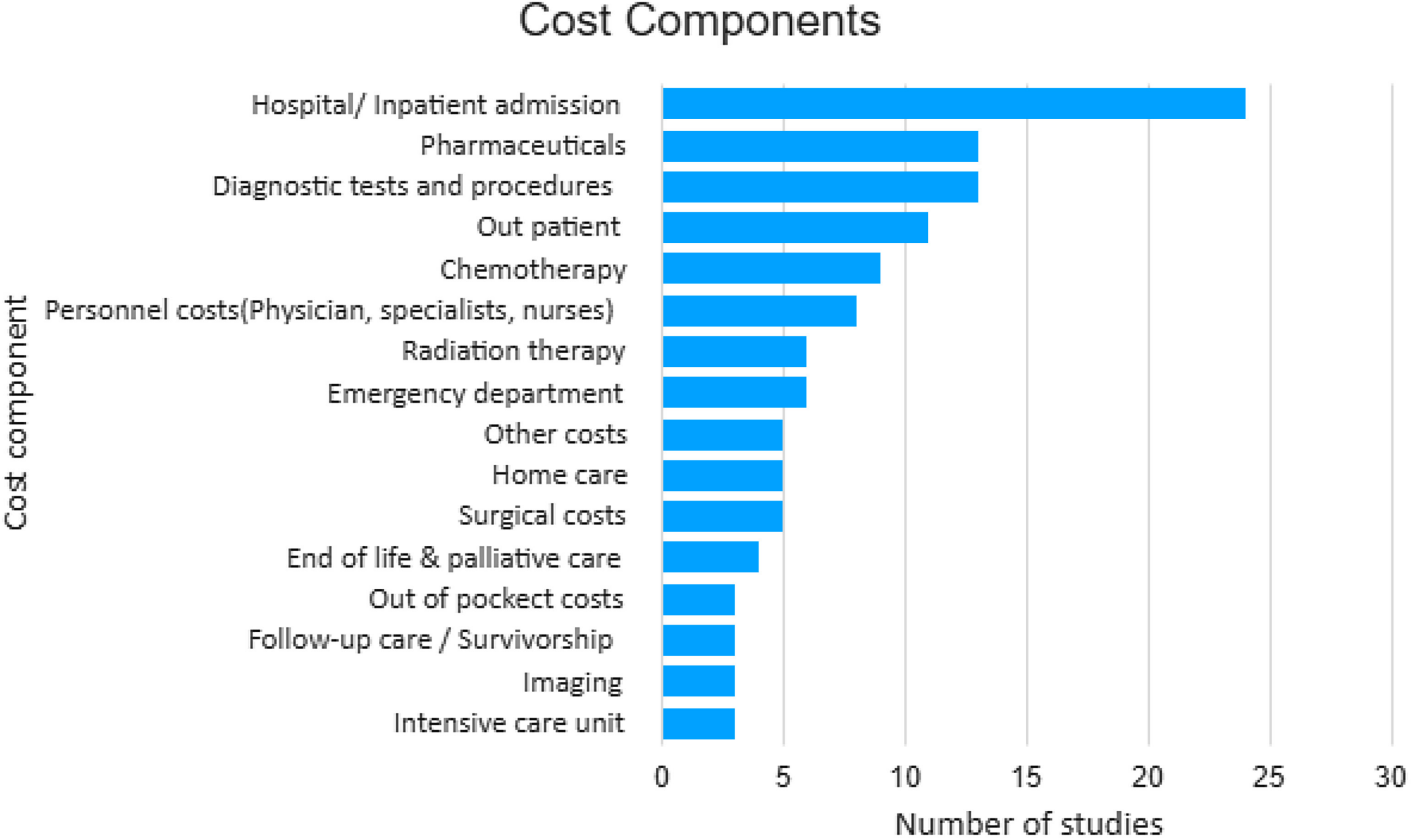
Number of studies reporting different types of costs (out of total of *N* = 30 included studies).

There was a wide mix of cost components mostly comprising hospital use or inpatient care (*n* = 21, 70%) as shown in Figure 2 and Table S5.^27^, ^29^, ^32^, ^33^, ^34^, ^43^, ^48^ The largest contributor to healthcare costs, according to nine studies,^33^, ^35^, ^37^, ^38^, ^39^, ^41^, ^50^, ^51^, ^54^ was hospitalizations, accounting for 45% to 93% of total costs (Table S4).

## 4 DISCUSSION

### 4.1 Summary of evidence

Most of the studies were from high‐income countries, predominantly the USA, with a broad range and mix of cost outcomes. In most studies, total costs were for children with cancer, related to hospital use, or specific to ALL. Findings in this review show healthcare costs were high for those diagnosed as children, those with leukemia, in the first year, and at end of life. Overall, costs related to hospitalization contributed to more than a third of the total healthcare costs.

Healthcare costs varied considerably across countries, according to this review. Since healthcare costs are a factor of volume and prices of health services, the variations from country‐specific policies for setting and regulating healthcare prices largely influence the healthcare costs in different settings.^56^, ^57^ In addition, factors such as the use of medical technologies,^58^ healthcare administration costs, and the lifestyle of the country's population influence health service use and costs.^57^ National healthcare financing and insurance policies, such as universal health care along with provider charges, influence healthcare utilization and costs.^59^, ^60^, ^61^ In the USA, for example, there is no Universal Health Coverage. Large segments of the population need private health insurance with government programs like Children Health Insurance Program (CHIPS) and Medicare limited to low‐income families. Unlike the USA, other countries have a public provision of essential services and medicines through tax‐funded universal healthcare or mandatory private health insurance. These systems increase access to essential health care services, protect individuals against financial risk, and increasing health service utilization and cost. Lastly, inconsistent conduct and study design elements, such as cost structures, cost perspectives, and other variables, could influence the magnitude of healthcare costs across studies, including those in a similar setting.^62^, ^63^

Our findings show healthcare costs during cancer care follow a U‐shape trend, highest during the initial year and end‐of‐life care, and lowest during the continuing phase. This is consistent with previous studies in the adult population which show a similar trend both during formal care^64^, ^65^, ^66^, ^67^, ^68^, ^69^ and informal care.^14^ In the study by Cheng Brian & Wangmo, provision of specialist palliative care was associated with lower cost compared to regular end‐of‐life care. We attribute this to the reduction in aggressive medication and treatments which are not in line with the patient's preferences and care needs.^70^, ^71^, ^72^ In the meta‐analysis by May et al, it was reported that palliative care is associated with reduced time in the hospital, which subsequently reduces the costs of healthcare.^71^

We noticed limited data on the healthcare costs for those who survive beyond 5‐year post cancer diagnosis. Previous research has highlighted a high morbidity burden caused by late effects of cancer, such as infectious complications, psychological conditions, chronic health issues, and secondary neoplasms^73^, ^74^, ^75^, ^76^ among the young cancer survivors. This information gap can therefore hinder effective healthcare planning, resource allocation and priority setting of health interventions for long‐term cancer survivors.

In line with our findings, national estimates from Australia on health system costs of cancer show leukemia as one of the costliest cancers among those aged below 20 years.^66^, ^77^ Literature for adult populations similarly show leukemia with the highest per‐person costs during follow‐up care^64^, ^66^ and was projected to cost 6.3% of the total global cost of cancers of 2020 to 2050^78^ despite lung, breast, prostate, and colorectal cancers being the most expensive in terms of overall cost burden. We can attribute this to the high cost of chemotherapy^79^ and high hospitalization resulting from the cytotoxic effect of leukemia treatment agents.

Our review reveals that hospitalization is the leading contributor to healthcare costs among children and AYA, which is not surprising. We can attribute this to several factors, including centralization of cancer treatment at hospital‐based centers for children and AYA due to specialist team needs which leads to longer hospital stays and increased costs. Because of their weak immune systems, children with cancer are more susceptible to severe illnesses, such as septicemia, fever, and neutropenia, resulting in increased hospitalizations during treatment.^80^, ^81^, ^82^, ^83^, ^84^

Although over 50% of new cancer cases are from low‐ and middle‐income (LMICs),^85^ there are several obstacles to the conduct of quality research in these countries such as limited resources, poor research infrastructure and general lack of comprehensive cancer registration and surveillance systems that generate reliable data.^85^

### 4.2 Study Limitations

Our study had limitations. First, we mainly included the direct cost of cancer to the healthcare system, which limited a comprehensive discussion of the cost burden of cancer to the individual and society. Second, because of our provider‐focused approach, we did not fully explore how diverse health insurance systems influence health care costs.

Due to the broad research question, the retrieved studies had heterogeneous outcomes, which restricted our capability to discuss costs across various study settings.

### 4.3 Clinical practice, policy, and research implications

Where possible, healthcare cost data collection and valuation methods should be standardized to support the comparability of cost outcomes across different settings.^86^ A national‐level healthcare cost data warehouse, suggested by Visscher et al., can facilitate data collection and linkage to administrative databases for health economic research at the country level.^87^ In addition, ensuring uniform methods for cost calculation, consistent discounting, presentation of cost outcomes and conduct of sensitivity analysis will reduce healthcare cost variations as recommended in a previous review.^63^

Regarding the cost of cancer, there is a need to put in place mechanisms that maximize value for money for cancer services to ensure sustainability of essential healthcare for patients with cancer. These include re‐evaluating the cost effectiveness of the current models of care and promote integration of palliative care at the end of life. This will facilitate prioritization of evidence‐based care, reduce on the wastage of health resources and consequently reduce the cost burden of children and AYA. Furthermore, provision of ongoing psychosocial care, promotion of healthy lifestyles could support patients to navigate through the challenging effects of cancer.^86^, ^88^

Better funding is necessary to improve childhood and AYA cancer registration in poorly monitored countries that will enable a more accurate and detailed assessment of healthcare costs incurred by health services and families and across different countries. This will lead to further research into the causes of cancers affecting children and young adults and determine whether there are modifiable risk factors that might be targeted to prevent these burdensome and costly cancers.

## 5 CONCLUSION

This review summarizes the current literature on healthcare costs for cancer among children, adolescents, and young adults. It reveals that cancer health resource costs vary depending on age, phase of care, cancer site, and types of resources. We know very little about the healthcare costs of cancer in low‐ and middle‐income countries and those attributed to the less common cancers.

## AUTHOR CONTRIBUTIONS

**Doreen Nabukalu:** Conceptualization (lead); data curation (lead); formal analysis (lead); methodology (lead); writing – original draft (lead); writing – review and editing (equal). **Louisa G. Gordon:** Conceptualization (equal); methodology (equal); supervision (equal); validation (equal); writing – review and editing (lead). **John Lowe:** Supervision (equal); writing – review and editing (equal). **Katharina M. D. Merollini:** Formal analysis (equal); methodology (equal); supervision (lead); validation (equal); writing – review and editing (equal).

## FUNDING INFORMATION

There was no external funding for this work. DN is supported by a PhD scholarship from the University of the Sunshine Coast.

## CONFLICT OF INTEREST STATEMENT

All authors declare no competing interests.

## Supporting information


Tables S1–S6.

Data S1.

## Data Availability

The data that supports the findings of this study are available from the corresponding author upon reasonable request.
