# Supplementary material for: Healthcare costs of cancer among children, adolescents, and young adults: A scoping review
Source: Cancer Med. 2024 Jan 12;13(3):e6925. doi: 10.1002/cam4.6925 (PMC10905233; doi:10.1002/cam4.6925)
Supplement: Supplementary file 1 — Tables S1–S6. [file CAM4-13-e6925-s001.docx]

**SUPPLEMENTARY TABLES AND FIGURES**

***Supplementary Table 1: Summary characteristics of N=30 included studies***

| **Study characteristics (N=30)** | **n** | % |
| --- | --- | --- |
| **Total number of studies** | 30 | 100 |
| **Year of publication** |  |  |
| 2012 | 1 | 3 |
| 2013 | 1 | 3 |
| 2014 | 1 | 3 |
| 2016 | 3 | 10 |
| 2017 | 6 | 20 |
| 2018 | 2 | 7 |
| 2019 | 2 | 7 |
| 2020 | 4 | 13 |
| 2021 | 5 | 17 |
| 2022 | 5 | 17 |
| **Cost calculation** |  |  |
| Bottom up | 27 | 90 |
| Top down | 2 | 7 |
| Mixed methods | 1 | 3 |
| **Cancer specific studies** |  |  |
| All cancers | 16 | 53.3 |
| Acute Lymphoblastic Leukemia (ALL) | 8 | 26.7 |
| All cancers except non melanoma skin cancers | 3 | 10.0 |
| All Caners except Leukemia | 1 | 3.3 |
| Solid organ or blood cancer | 1 | 3.3 |
| Bone sarcomas | 1 | 3.3 |
| **Age group** |  |  |
| 0 - 39 Total | 4 | 13 |
| 0-14 Total | 4 | 13 |
| 0-19 Total | 16 | 53 |
| 15 - 19 Total | 1 | 3 |
| 15 - 39 Total | 5 | 17 |
| **Study design** |  |  |
| External validation study | 1 | 3 |
| Cost effective analysis. | 2 | 7 |
| Cost-analysis | 2 | 7 |
| Retrospective cohort | 19 | 63 |
| Cross sectional analysis | 3 | 10 |
| Panel survey | 3 | 10 |

***Supplementary Table 2: Study Setting***

| **Study setting** | **n (%)** | **Income Status** | **Main form of health Insurance system.** |
| --- | --- | --- | --- |
| USA | 12(40) | High income | Private health insurance |
| Netherlands | 2(7) | High income | Mandatory private health insurance |
| Canada | 5(17) | High income | Publicly funded health care system |
| Australia | 2(7) | High income | Publicly funded health care system |
| Chile | 1(3) | High income | Publicly funded health care system |
| China | 1(3) | Upper middle income | Publicly funded basic medical insurance |
| Egypt | 1(3) | Upper middle income | Publicly funded health care system |
| France | 1(3) | High income | Mandatory statutory insurance provided by non-competitive insurers |
| India | 1(3) | Upper middle income | Publicly funded health care system |
| Korea | 1(3) | High income | Publicly funded health care system |
| Mexico | 1(3) | Upper middle income | Publicly funded health care system |
| Spain | 1(3) | High income | Publicly funded health care system |
| Thailand | 1(3) | Upper middle income | Publicly funded health care system |

***Supplementary Table 3: Cancer specific costs***

| **Study** | **Country** | **Units of measure** | **Health care costs by type of cancer (2022 US Dollars)** | | | | | | | |
| --- | --- | --- | --- | --- | --- | --- | --- | --- | --- | --- |
|  |  |  | **0-14 years /0-19** | | | | **15-19 years** | | | |
|  |  |  | **Leukemia** | **Brain / CNS** | **Lymphoma** | **Bone & articular cartilage** | **Leukemia** | **Brain / CNS** | **Lymphoma** | **Bone & articular cartilage** |
| de Oliveira 2017 | Canada | Mean total costs per weighted case | $149,285 | $97,332 | $101,792 |  | $157,029 | $59,859 | $54,272 | $106,153 |
| Oliveira 2017 | Canada | Mean total costs per weighted case | $152,533 | $112,443 | $94,538 |  | $166,670 | $72,179 | $49,890 | $118,269 |
| McBride 2020 | Canada (British Columbia) | Mean net total cost per patient | $103,731 | $81,695 | $79,060 |  |  |  |  |  |
| McBride 2020 | Canada (Ontario) | Mean net total cost per patient | $151,269 | $111,406 | $93,323 |  |  |  |  |  |
| Chae 2020 | Korea | Mean total per person | $53,279 | $36,140 | $37,160 | $51,865 |  |  |  |  |
| Nathan 2019 | Canada | Median annual total cost per person |  |  |  |  | $149,045 | $46,893 | $62,962 | $183,419 |
| Borrescio-Higa 2018 | Chile | Average annual medical costs in 2018 | $50,133 | $39,839 | $37,366 |  |  |  |  |  |
| **Hospitalisation Costs** |  |  |  |  |  |  |  |  |  |  |
| Soliman 2021 | Egypt | Median total costs per patient | $25,247 | $9,077 | $8,022 | $23,690 |  |  |  |  |
| Tan 2022 | Australia | mean annual total cost per patient | $101,920 | $89,138 | $82,932 | $99,391 |  |  |  |  |

*S****upplementary Table 4: Hospital related costs reported in selected studies (in US$)***

|  | Total medical costs | Hospital use related costs | **percentage (%) of total cost** |
| --- | --- | --- | --- |
| Bejarano-Quisoboni 2022 | $4,255 | $1,919 | 45.1 |
| Borrescio-Higa 2018 | $32,287 | $29,888 | 92.6 |
| Chae 2020 | $36,799 | $28,321 | 77.0 |
| de Oliveira 2017 | $173,202 | $143,762 | 83.0 |
| Oliveira 2017 | $139,453 | $106,660 | 76.5 |
| Gupta 2021 | $238,800 | $117,800 | 49.3 |
| McBride 2020 | $138,161 | $106,660 | 77.2 |
| McGrady 2017 | $384,464 | $310,799 | 80.8 |
| Mueller 2017 | $1,930 | $1,010 | 52.3 |

***Supplementary Table 5: Type of cost reported in N=30 included studies***

|  | **Total medical costs** | **Hospitalisation** | | | | | | | | | | **Out of pocket** | **Treatment specific costs** | | | | **Personnel** | Indirect | Societal |
| --- | --- | --- | --- | --- | --- | --- | --- | --- | --- | --- | --- | --- | --- | --- | --- | --- | --- | --- | --- |
|  |  | General Hospital use | Out patient | Inpatient | Intensive care unit | Emergency department | Diagnostic tests & procedures | Imaging | Pharmaceuticals | Follow-up | End of life |  | Chemotherapy | Surgical | Home care | Radiation therapy |  |  |  |
| Abdelhadi 2022 | 1 |  |  |  |  |  |  |  |  |  |  |  |  |  |  |  |  |  |  |
| Abdelhadi 2022 | 1 |  |  |  |  |  |  |  |  |  |  |  |  |  |  |  |  |  |  |
| Audino 2012 |  | 1 |  |  |  |  | 1 | 1 | 1 |  |  |  |  |  |  |  |  |  |  |
| Bejarano-Quisoboni 2022 |  | 1 |  |  |  |  | 1 |  | 1 | 1 |  |  |  |  |  |  | 1 | 1 | 1 |
| Benedict 2021 | 1 |  |  |  |  |  |  |  |  | 1 |  |  |  |  |  |  |  |  |  |
| Borrescio-Higa 2018 | 1 |  | 1 | 1 |  |  |  |  |  |  |  | 1 |  |  |  |  |  |  |  |
| Chae 2020 | 1 |  | 1 | 1 |  |  |  |  |  |  |  |  | 1 | 1 |  | 1 |  |  |  |
| Cheng Brian & Wangmo 2020 |  | 1 |  |  |  |  |  |  |  |  | 1 |  |  |  |  |  |  |  |  |
| de Oliveira 2017 | 1 |  | 1 | 1 |  | 1 | 1 |  | 1 |  |  |  | 1 |  | 1 | 1 | 1 |  |  |
| Oliveira 2017 |  |  | 1 | 1 |  | 1 | 1 |  | 1 |  | 1 |  | 1 |  | 1 | 1 | 1 |  |  |
| Ghatak 2016 | 1 | 1 |  |  |  |  | 1 |  | 1 |  |  | 1 |  |  |  |  |  | 1 | 1 |
| Gupta 2021 | 1 |  |  |  |  |  |  |  |  |  |  |  | 1 |  |  |  |  |  |  |
| Guy 2014 | 1 |  | 1 | 1 |  |  |  |  |  |  |  | 1 |  |  |  |  |  |  | 1 |
| Haeusler 2018 | 1 |  |  |  | 1 | 1 | 1 |  | 1 |  |  |  |  | 1 | 1 |  |  |  |  |
| Jaime-Pérez 2017 |  | 1 |  |  |  |  |  |  |  |  |  |  |  |  |  |  |  |  |  |
| Kloos 2018 | 1 |  | 1 | 1 | 1 |  | 1 |  | 1 |  |  |  | 1 | 1 | 1 |  |  |  |  |
| Lekshminarayanan 2018 |  | 1 |  |  |  |  |  |  |  |  |  |  |  |  |  |  |  |  |  |
| McBride 2020 | 1 |  |  | 1 |  |  | 1 |  |  |  | 1 |  | 1 |  |  | 1 | 1 |  |  |
| McGrady 2017 | 1 | 1 | 1 | 1 |  | 1 |  |  |  |  |  |  |  |  |  |  |  |  |  |
| Mueller 2017 | 1 | 1 | 1 |  |  |  | 1 | 1 | 1 |  |  |  |  |  |  |  | 1 |  |  |
| Mueller 2016 |  | 1 |  |  |  |  |  |  |  |  |  |  |  |  |  |  |  |  |  |
| Nathan 2019 | 1 | 1 | 1 | 1 |  | 1 | 1 |  |  |  |  |  | 1 |  | 1 | 1 | 1 |  |  |
| Maria 2020 |  | 1 | 1 |  | 1 |  | 1 |  | 1 | 1 | 1 |  |  |  |  |  | 1 |  |  |
| Kaul 2016 |  | 1 |  |  |  |  |  |  |  |  |  |  |  |  |  |  |  |  |  |
| Soliman 2021 |  |  |  |  |  |  | 1 | 1 | 1 |  |  |  |  | 1 |  | 1 | 1 |  |  |
| Sruamsiri 2020 |  |  |  |  |  |  |  |  | 1 |  |  |  | 1 |  |  |  |  |  |  |
| Tan 2022 |  | 1 |  |  |  | 1 |  |  |  |  |  |  |  |  |  |  |  |  |  |
| Taparra 2022 |  | 1 |  |  |  |  |  |  |  |  |  |  |  |  |  |  |  |  |  |
| Tong 2013 | 1 |  | 1 | 1 |  |  | 1 |  | 1 |  |  |  | 1 |  |  |  |  |  |  |
| Zhou 2021 | 1 |  |  |  |  |  |  |  | 1 |  |  |  |  | 1 |  |  |  |  |  |
| Total | 17 | 14 | 11 | 10 | 3 | 6 | 13 | 3 | 13 | 3 | 4 | 3 | 9 | 5 | 5 | 6 | 8 | 2 | 3 |

***Supplementary Table 6: CHEERS reporting checklist applied to N=30 selected studies***

| Study | Abstract | Background & objectives | Study population | Setting and location | Perspective | Time horizon | Measurement and valuation of resources and costs | Currency, price date, and conversion | Analytics and assumptions | Summary of main results | Study findings, limitations, generalisability, & current knowledge | Source of funding | Conflicts of interest | Total | % score |
| --- | --- | --- | --- | --- | --- | --- | --- | --- | --- | --- | --- | --- | --- | --- | --- |
| Abdelhadi 2022 | 1 | 1 | 1 | 1 | 0 | 1 | 1 | 1 | 1 | 1 | 1 | 0.5 | 1 | 11.5 | 88 |
| Abdelhadi 2022 | 1 | 1 | 1 | 1 | 0 | 1 | 1 | 1 | 1 | 1 | 1 | 0.5 | 1 | 11.5 | 88 |
| Audino 2013 | 1 | 1 | 1 | 1 | 0 | 1 | 1 | 1 | 1 | 1 | 1 | 0 | 1 | 11 | 84 |
| Bejarano-Quisoboni 2022 | 1 | 1 | 1 | 1 | 1 | 1 | 1 | 1 | 1 | 1 | 1 | 1 | 1 | 13 | 100 |
| Benedict 2021 | 0.5 | 1 | 1 | 1 | 0 | 1 | 1 | 0.5 | 1 | 1 | 1 | 0.5 | 1 | 10.5 | 81 |
| Borrescio-Higa 2018 | 0.5 | 1 | 1 | 1 | 0 | 1 | 1 | 1 | 1 | 1 | 1 | 1 | 1 | 11.5 | 88 |
| Chae 2020 | 0.5 | 1 | 1 | 1 | 1 | 1 | 1 | 1 | 1 | 1 | 1 | 1 | 1 | 12.5 | 96 |
| Cheng Brian & Wangmo 2020 | 1 | 1 | 1 | 1 | 0 | 1 | 1 | 1 | 1 | 1 | 1 | 0 | 1 | 11 | 84 |
| Oliveira 2017 | 1 | 1 | 1 | 1 | 1 | 1 | 1 | 1 | 1 | 1 | 1 | 1 | 1 | 13 | 100 |
| de Oliveira 2017 | 1 | 1 | 1 | 1 | 1 | 1 | 1 | 1 | 1 | 1 | 1 | 1 | 1 | 13 | 100 |
| Ghatak 2016 | 1 | 1 | 1 | 1 | 1 | 1 | 1 | 1 | 0.5 | 1 | 1 | 0 | 1 | 11.5 | 88 |
| Mueller 2016 | 0.5 | 1 | 1 | 1 | 0 | 1 | 0.5 | 1 | 1 | 1 | 0.5 | 0 | 1 | 9.5 | 73 |
| Mueller 2017 | 1 | 1 | 1 | 1 | 0 | 1 | 1 | 0.5 | 0.5 | 1 | 0.5 | 0 | 1 | 9.5 | 73 |
| Gupta 2021 | 1 | 1 | 1 | 1 | 1 | 1 | 1 | 1 | 1 | 1 | 1 | 1 | 1 | 13 | 100 |
| Guy 2014 | 0.5 | 1 | 1 | 0.5 | 0 | 1 | 1 | 1 | 1 | 1 | 1 | 0 | 0 | 9 | 69 |
| Haeusler 2018 | 1 | 1 | 1 | 1 | 0 | 1 | 1 | 1 | 1 | 1 | 1 | 1 | 1 | 12 | 92 |
| Jaime-Pérez 2017 | 1 | 1 | 1 | 1 | 0 | 1 | 1 | 1 | 1 | 1 | 1 | 0 | 1 | 11 | 84 |
| Kloos 2019 | 1 | 1 | 1 | 1 | 1 | 1 | 1 | 1 | 1 | 1 | 1 | 1 | 1 | 13 | 100 |
| Lekshminarayanan 2018 | 1 | 1 | 1 | 1 | 0 | 1 | 1 | 0.5 | 1 | 1 | 1 | 0 | 1 | 10.5 | 81 |
| McBride 2020 | 1 |  | 1 | 1 | 0 | 1 | 1 | 1 | 1 | 1 | 1 | 1 | 1 | 11 | 85 |
| McGrady 2017 | 1 | 1 | 1 | 1 | 0 | 1 | 0.5 | 1 | 1 | 1 | 1 | 1 | 1 | 11.5 | 88 |
| Nathan 2019 | 1 | 1 | 1 | 1 | 1 | 1 | 1 | 1 | 1 | 1 | 1 | 1 | 1 | 13 | 100 |
| Maria 2020 | 1 | 1 | 1 | 0 | 1 | 1 | 1 | 1 | 1 | 1 | 1 | 1 | 1 | 12 | 92 |
| Kaul 2016 | 0.5 | 1 | 1 | 1 | 1 | 1 | 1 | 1 | 1 | 1 | 1 | 1 | 1 | 12.5 | 96 |
| Soliman 2021 | 1 | 1 | 1 | 1 | 1 | 1 | 1 | 1 | 1 | 1 | 1 | 1 | 1 | 13 | 100 |
| Sruamsiri 2020 | 1 | 1 | 1 | 1 | 0 | 1 | 1 | 1 | 1 | 1 | 1 | 1 | 1 | 12 | 92 |
| Tan 2022 | 1 | 1 | 1 | 1 | 1 | 1 | 1 | 1 | 1 | 1 | 1 | 1 | 1 | 13 | 100 |
| Taparra 2022 | 1 | 1 | 1 | 1 | 0 | 1 | 1 | 0.5 | 1 | 1 | 1 | 1 | 1 | 11.5 | 88 |
| Tong 2013 | 0.5 | 1 | 1 | 0.5 | 0 | 1 | 1 | 1 | 1 | 1 | 1 | 0.5 | 0 | 9.5 | 73 |
| Zhou 2021 | 1 | 1 | 1 | 1 | 0 | 1 | 1 | 1 | 1 | 1 | 1 | 1 | 1 | 12 | 92 |
| **Total score** | 26.5 | 29 | 30 | 28 | 12 | 30 | 29 | 28 | 29 | 30 | 29 | 20 | 28 |  |  |
| **Percentage score** | 88 | 97 | 100 | 93 | 40 | 100 | 97 | 93 | 97 | 100 | 97 | 67 | 93 |  |  |
