# Supplementary material for: Healthcare costs of cancer among children, adolescents, and young adults: A scoping review
Source: Cancer Med. 2024 Jan 12;13(3):e6925. doi: 10.1002/cam4.6925 (PMC10905233; doi:10.1002/cam4.6925)

**HEALTHCARE COSTS OF CANCER AMONG CHILDHOOD, ADOLESCENTS, AND YOUNG ADULT CANCER SURVIVORS**

**A SCOPING REVIEW SEARCH STRATEGIES**

1. Medline Search strategy


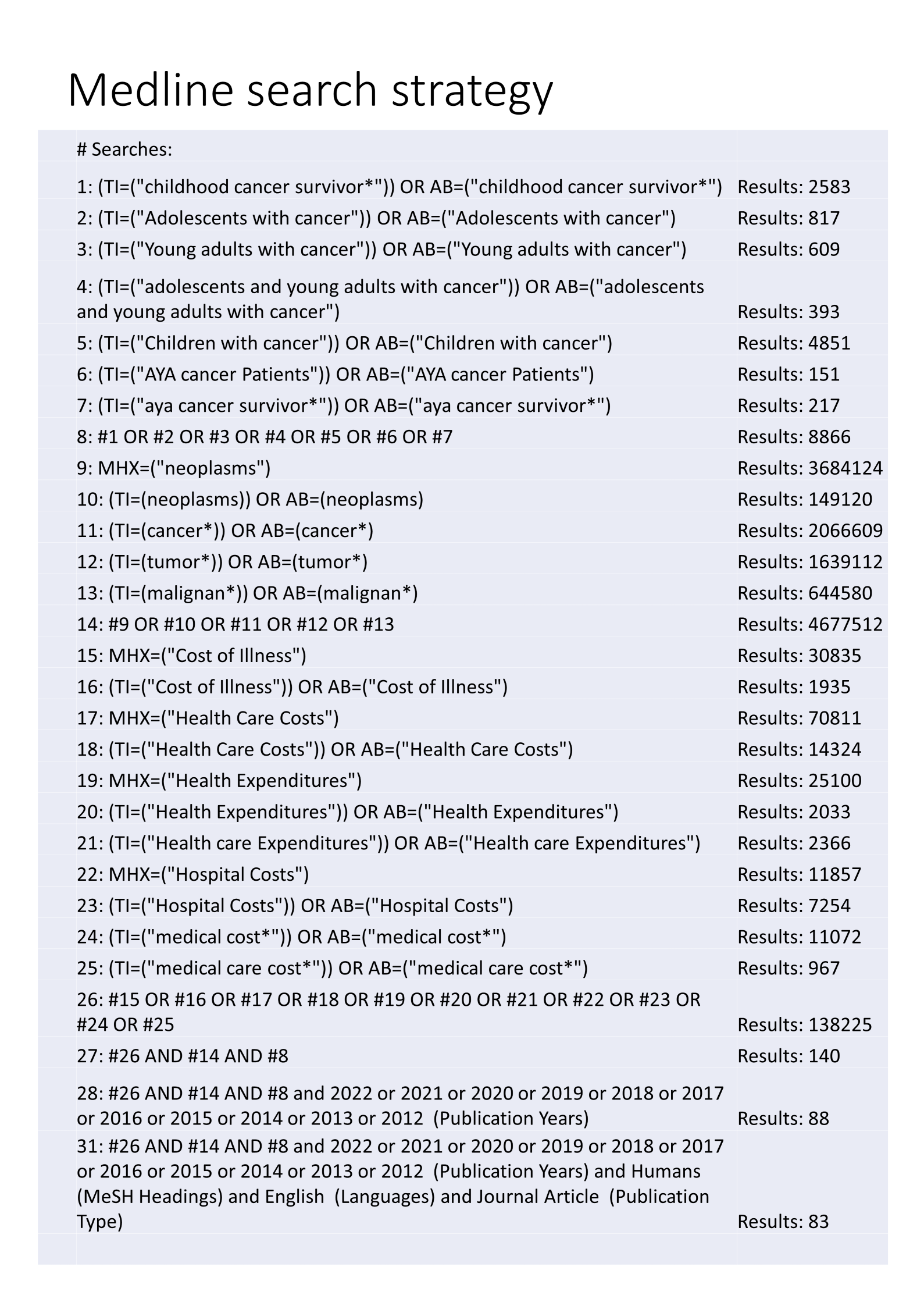


1. PubMed Search strategy


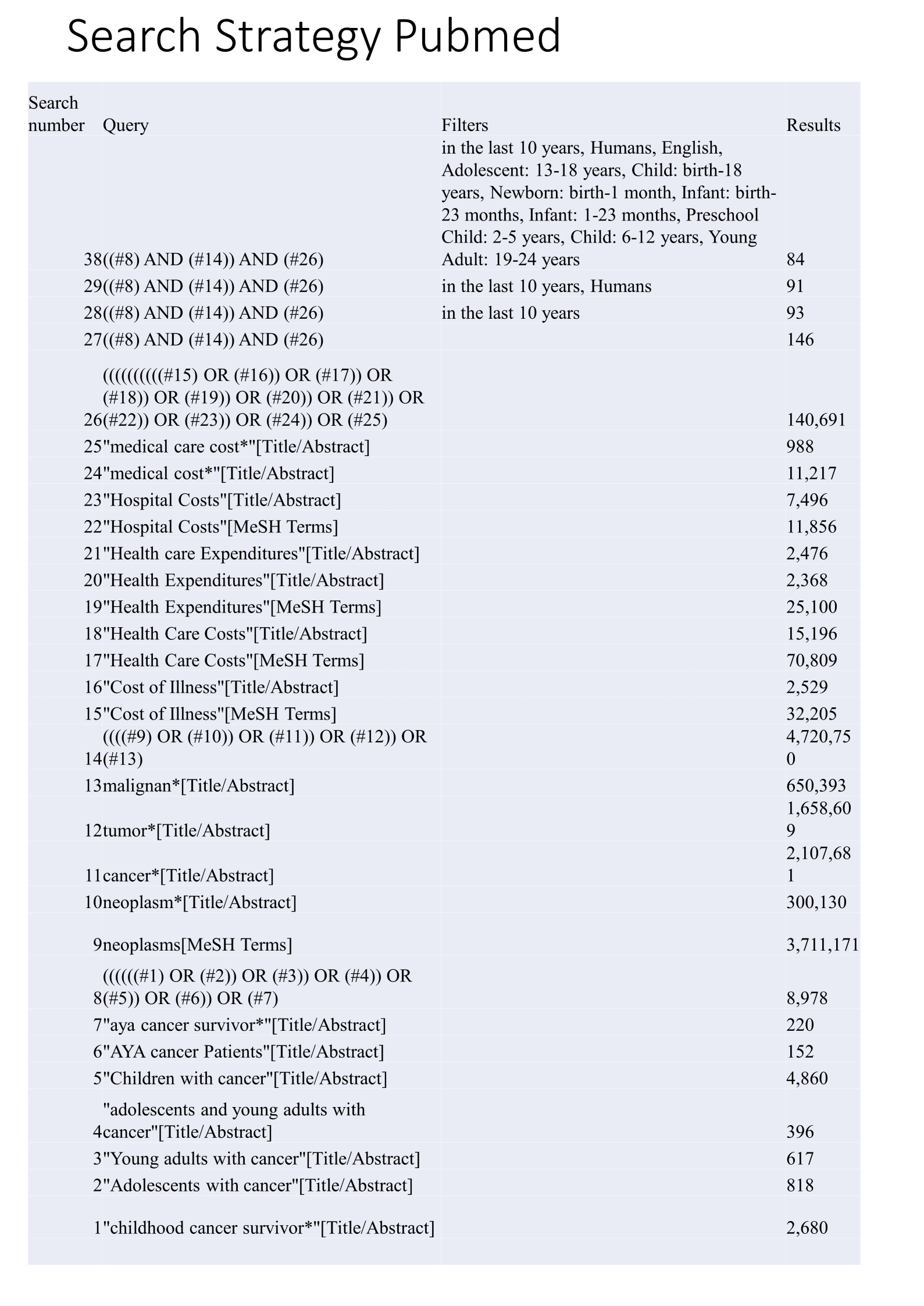


1. CINHAL Search strategy


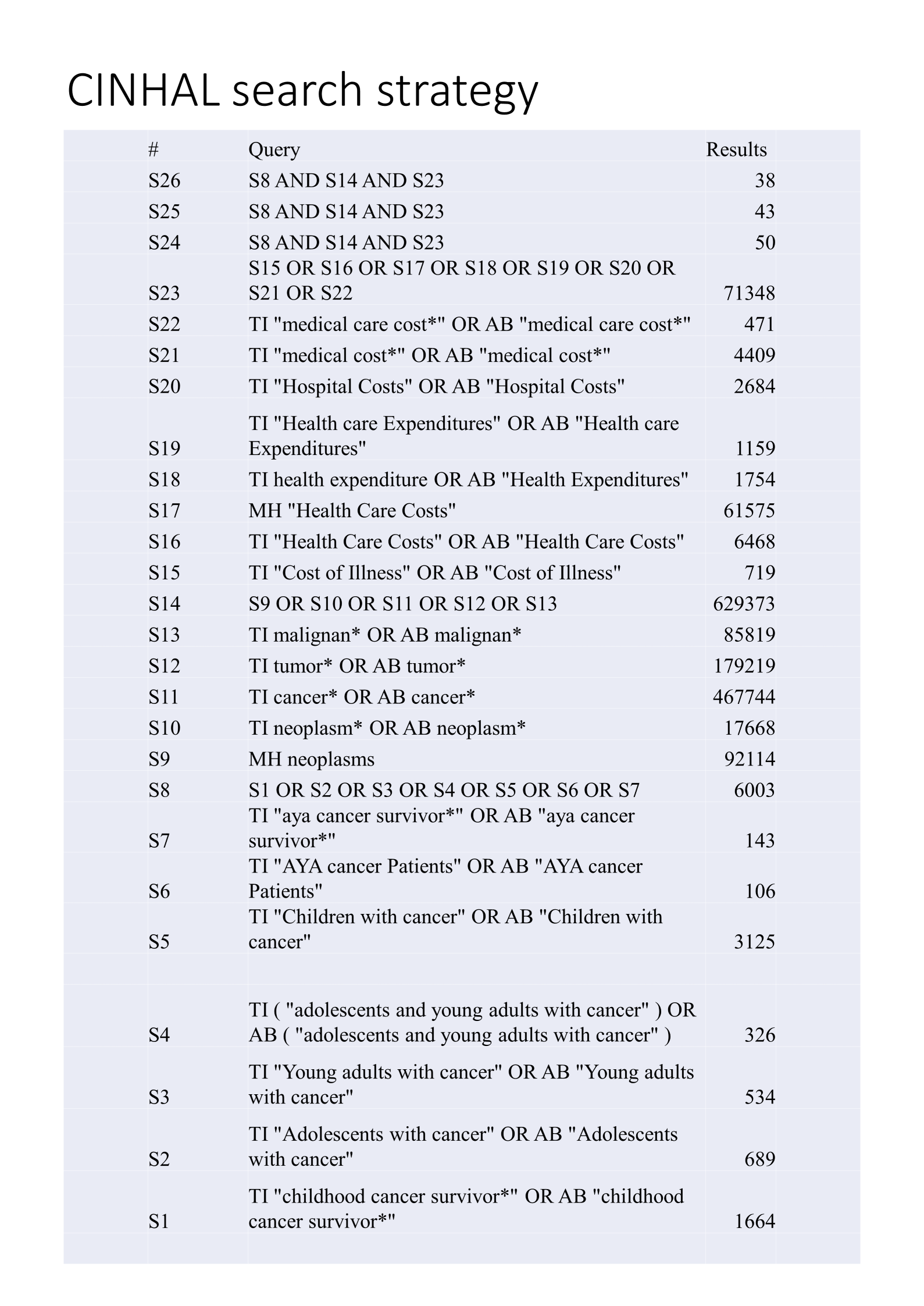


1. Scopus Search strategy


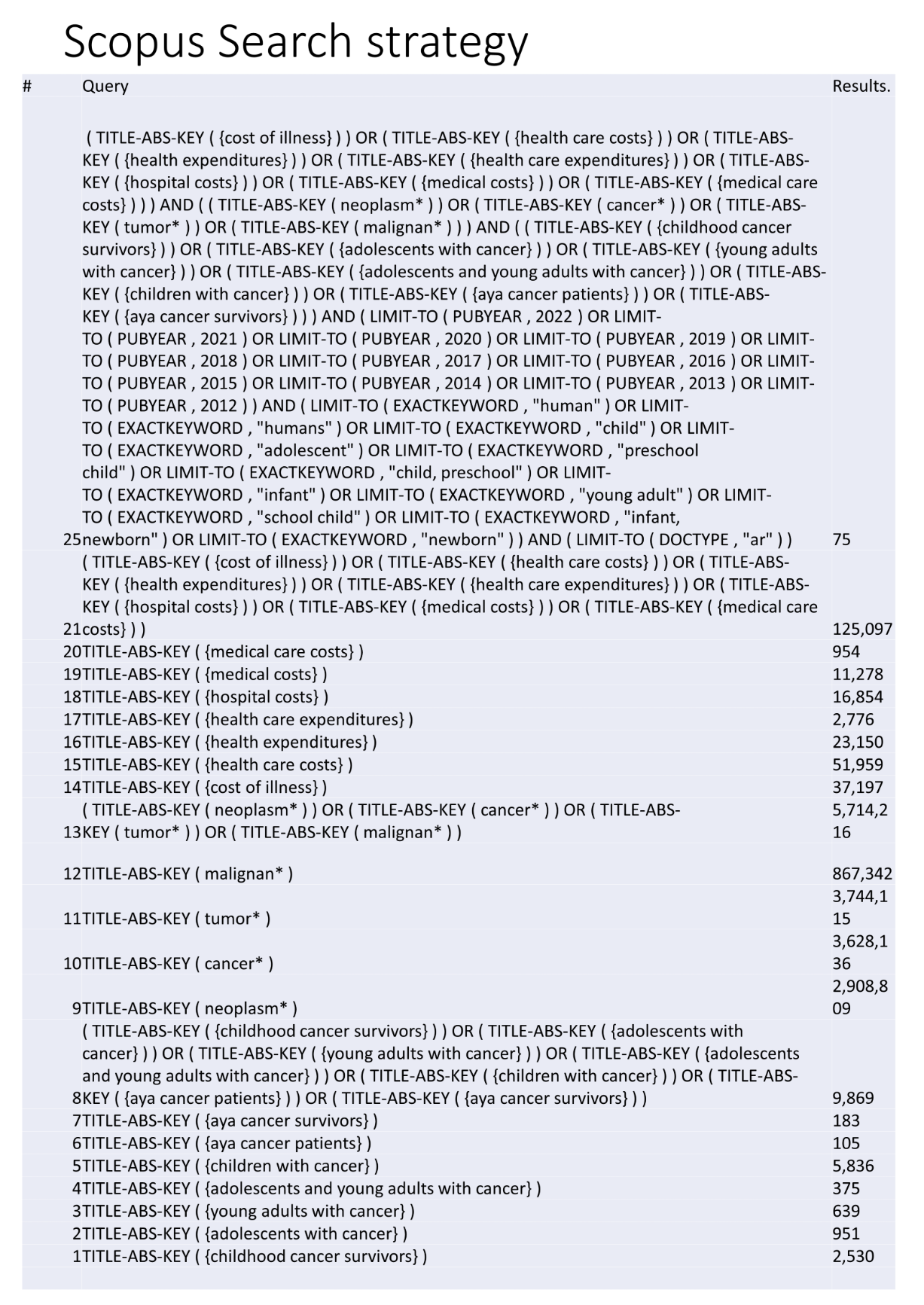

Supplement: Supplementary file 2 — Data S1. [file CAM4-13-e6925-s002.docx]
